# Hyperostosis Frontalis Interna in a Dry Indian Human Skull: A Case Report

**DOI:** 10.7759/cureus.34645

**Published:** 2023-02-05

**Authors:** Suranjana Banik, Manisha Gaikwad, Nerbadyswari Deep

**Affiliations:** 1 Anatomy, All India Institute of Medical Sciences, Bhubaneswar, IND; 2 Radiology, All India Institute of Medical Sciences, Bhubaneswar, IND

**Keywords:** depression, parkinsonism, frontal bone, schizophrenia, hyperostosis

## Abstract

Hyperostosis Frontalis Interna (HFI), a condition that has been sparsely explained till now, is a benign, asymptomatic, and irregular thickening of the endocranium of the frontal bone. It is found to be predominantly present in post-menopausal women during incidental X-ray or CT/MRI of the skull. The prevalence of HFI is documented in different populations, but in the Indian population, it is comparatively rare. Thus, we discuss a serendipitous finding of HFI in an Indian skull. This rare variation was noted in dry Indian human skulls. Gross features of the skull were noted, and it was an adult female skull. The area was decalcified, paraffin-embedded, and stained with Haematoxylin and Eosin. The skull bone was also subjected to plain X-ray/CT investigation. The X-ray skull of 50+ year female type features in anteroposterior and lateral view showed widening of the diploic spaces 8-10 mm with ill-defined hyperdense areas in the frontal region. Changes in computed tomography were noted. HFI often has nonspecific and benign symptoms. However, in severe cases, widespread clinical implications starting from headache, motor aphasia, parkinsonism, and depression can occur, and thus we all should be aware of it.

## Introduction

Hyperostosis Frontalis Interna (HFI) is a sparsely explained, benign, asymptomatic, and irregular thickening of the endocranium of the frontal bone. It is predominantly present in post-menopausal women during incidental X-ray or CT/MRI of the skull [1]. Morgagni first described Hyperostosis Frontalis Interna in the year 1769 when he coined this descriptive term to denote a specific variety of bone acceleration on the inner table of the frontal bone with midline sparing [2]. It may be present as single or multiple bilateral nodules on the inner lamina without damaging diploe and external bony lamina [3]. This can be appreciated better in CT/MRI and X-rays in living subjects and cadavers during dissection (1955). Hyperostosis Frontalis Interna was initially thought to be associated with Morgagni's syndrome (HFI, obesity, virilism), Stewart-Morel syndrome (HFI, obesity, neuropsychiatric disorders), Troell-Junet syndrome (HFI, acromegaly, goiter, diabetes) but it is now considered to be present as a separate entity too [4,5]. The prevalence of HFI is documented in different populations, but in the Indian population, it is comparatively rare. Thus, we discuss a serendipitous finding of HFI in a dry Indian skull. 

## Case presentation

**Figure 1:**
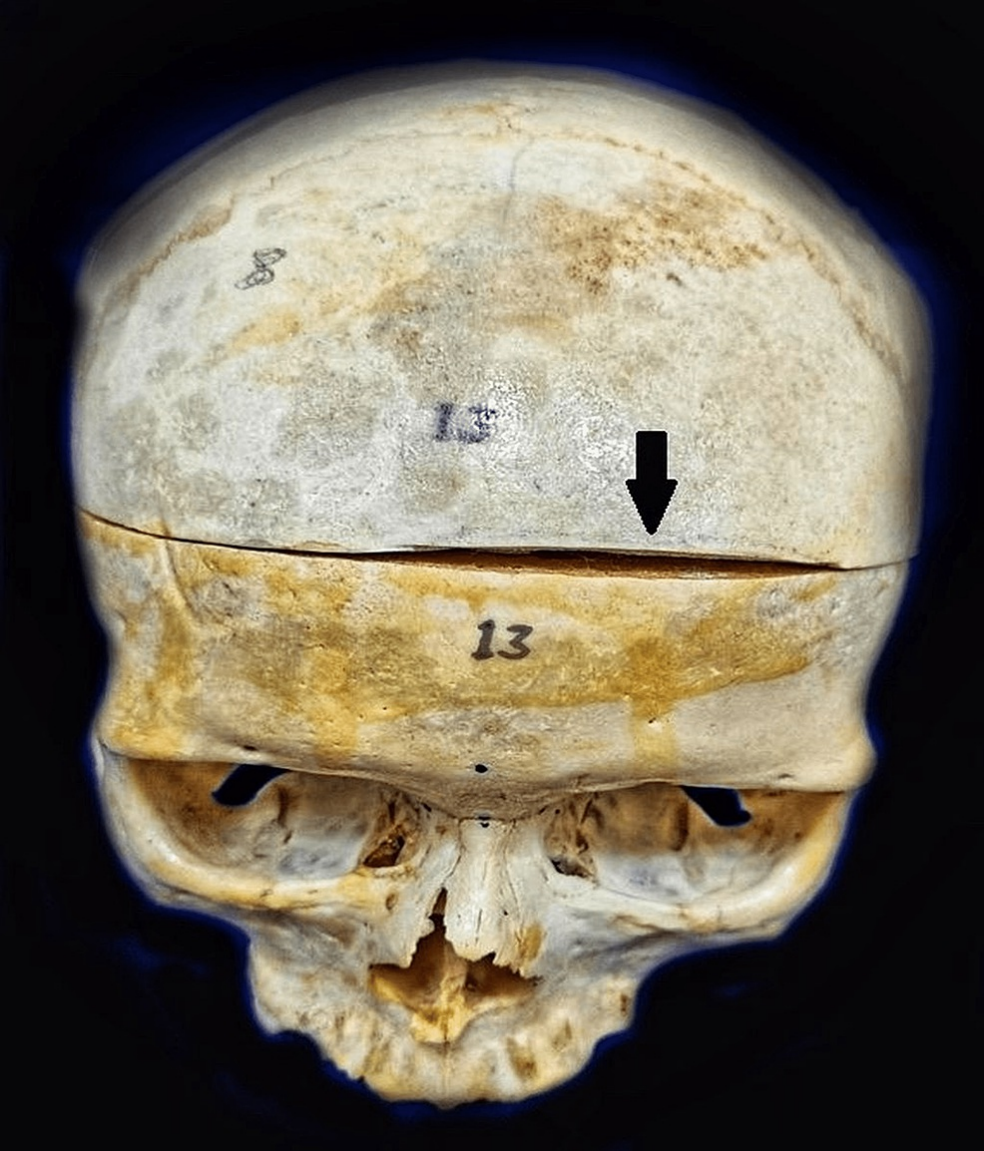
The external surface of the skull shows the normal appearance of the bones of the skull. The black arrow denotes the bony tissue taken from the area for decalcification and tissue processing.

**Figure 2:**
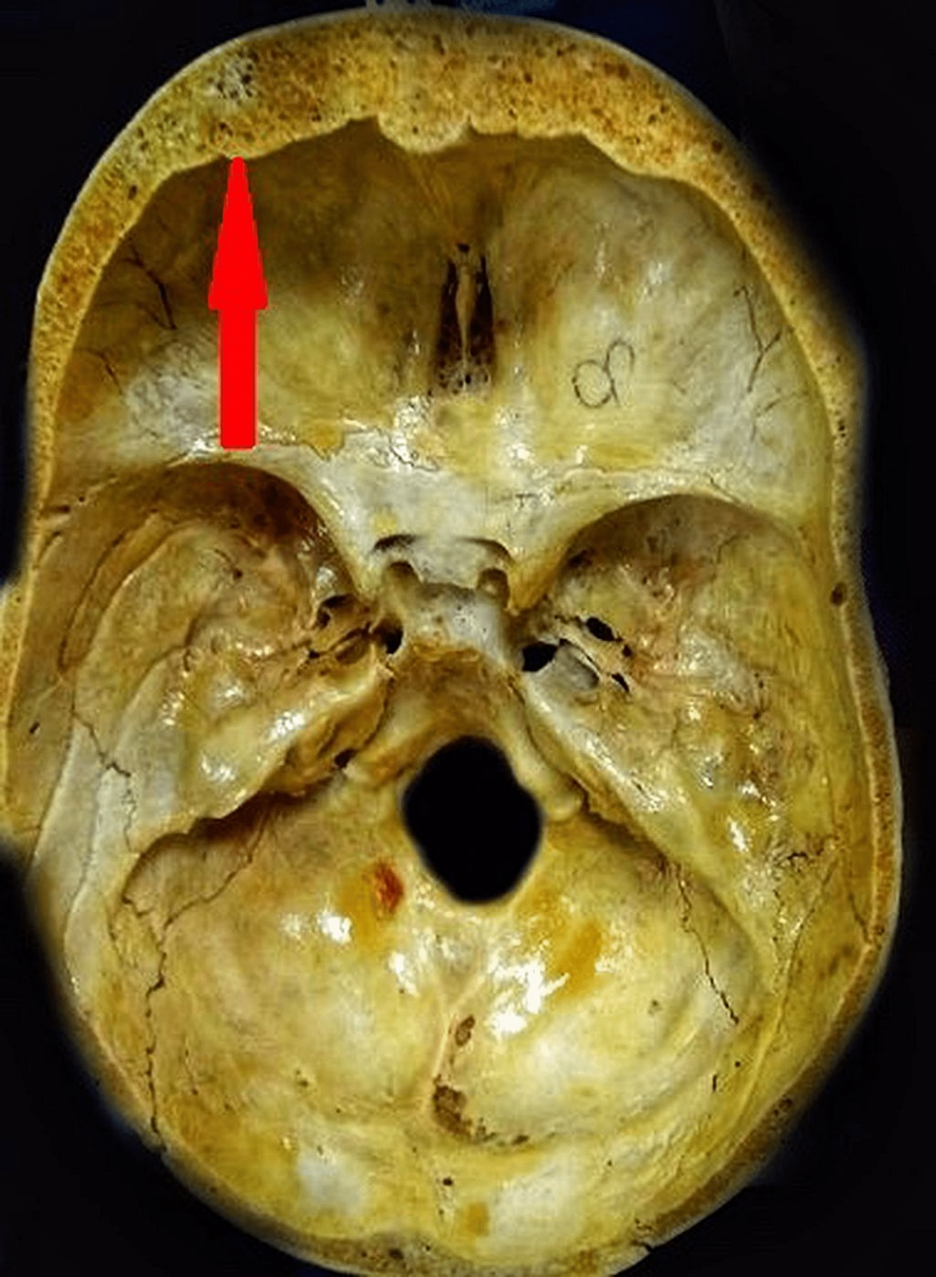
The irregular thickness of the frontal bone with an increase in distance between the outer and inner table seen in the region of frontal bone shown by the red arrow is 13.44 mm by digital vernier caliper, YAMAYO.

**Figure 3:**
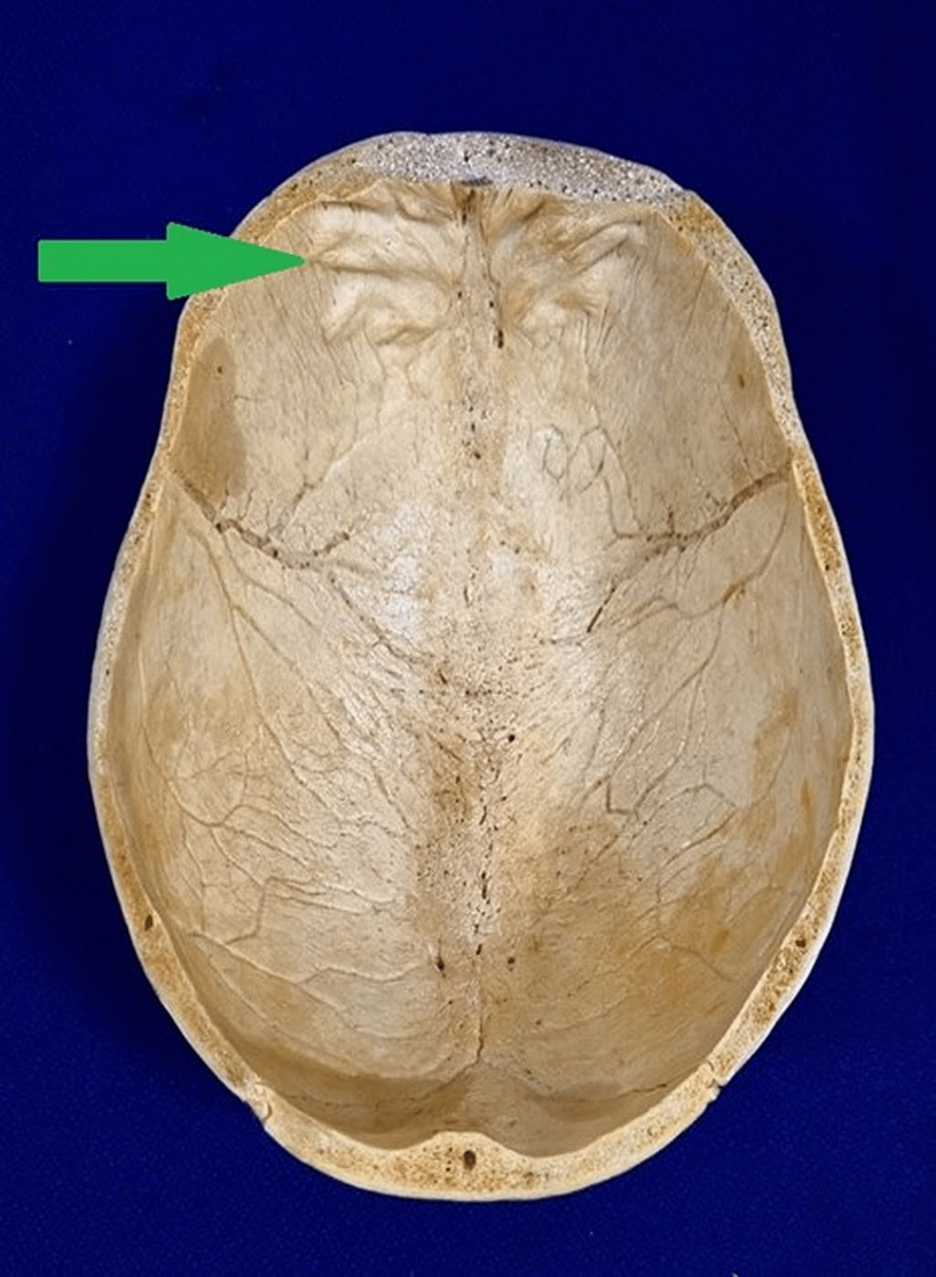
The internal surface of the frontal bone shows the multiple smooth bony ridges with midline sparing shown by a green arrow.

This rare variation was noted in a dry Indian human skull in the Department of Anatomy during osteology class for 1st-year MBBS students at our institute. Gross features of the skull were noted, and it was an adult female skull as per the osteological features and morphometric measurements. The bony chip of the affected area was decalcified, paraffin-embedded, and stained with Hematoxylin & Eosin. The skull bone was also subjected to plain X-ray/CT investigation. The exterior of the skull appeared normal, but the inner aspect showed unusual irregular thickening and elevated nodular areas along the endocranial surface of the frontal bone shown by the red arrow is 13.44 mm by the digital vernier caliper from YAMAYO shown in Figure 1-3.

**Figure 4:**
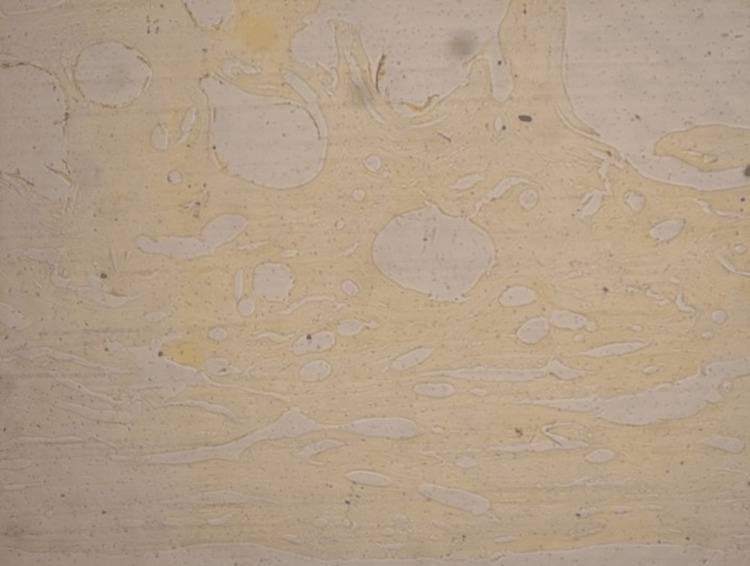
The decalcified and the unstained paraffin-embedded tissue section showing the classical cancellous type of bony pattern in the photomicrograph.

**Figure 5:**
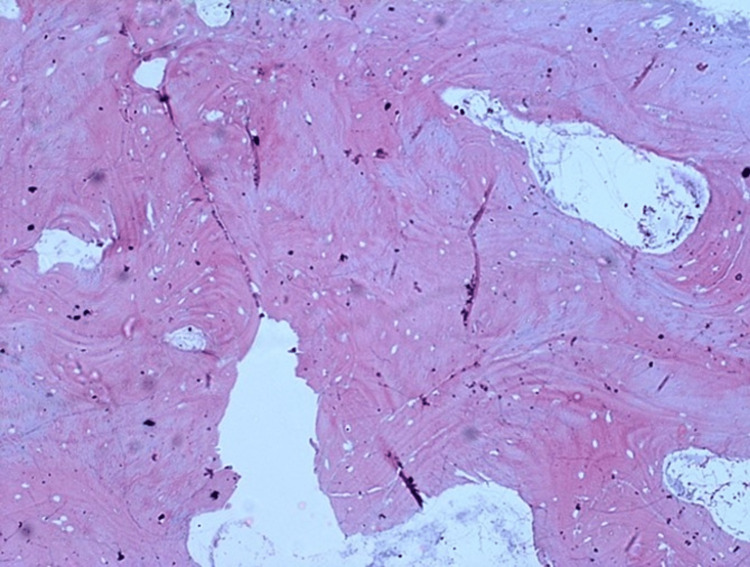
The decalcified, paraffin-embedded & H & E-stained section of the bony piece shows the presence of compact bone histologically. It could be appreciated along the inner and outer tables of the skull, with a classical lamellar pattern. However, the central part, which was more of a cancellous type, was with thickened and irregular bony trabeculae.

**Figure 6:**
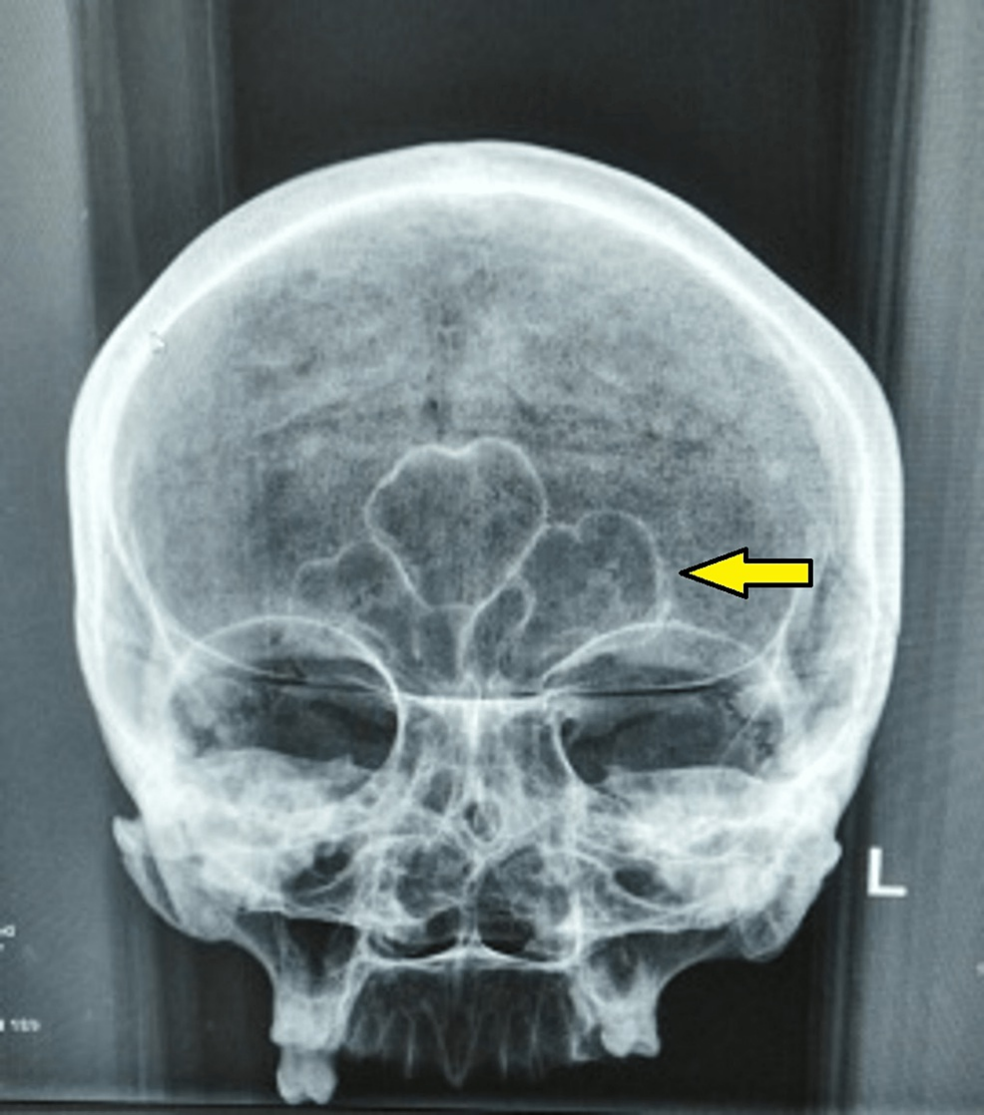
The plane X-ray skull of 50 years + female in AP shows frontal air sinuses marked by a yellow arrow.

**Figure 7:**
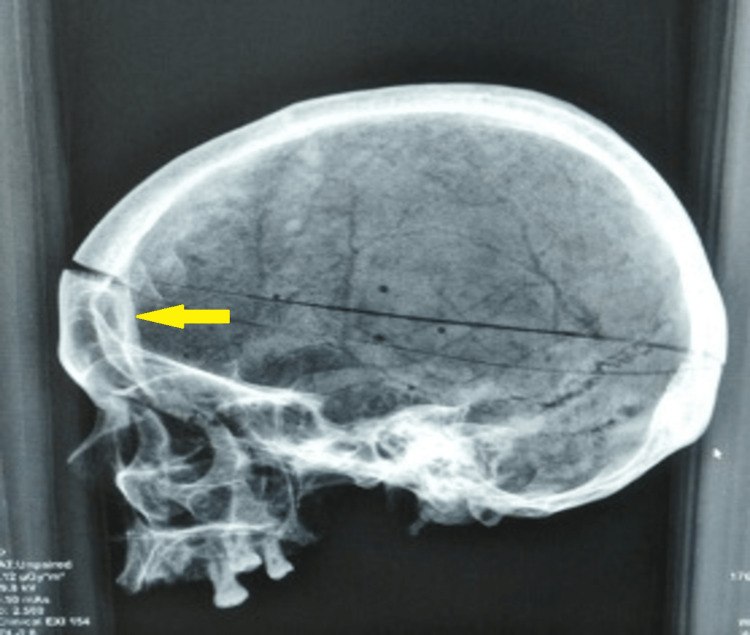
Widening of the diploic spaces 8-10 mm with ill-defined hyperdense areas in the frontal region shown by a yellow arrow. Widening of the diploic spaces 8-10 mm with ill-defined hyperdense areas in the frontal region.

The skull tissue was decalcified, and paraffin embedding was done, as shown in Figure 4. H & E staining of a section of the bony piece showed the increase in the thickness of the trabecular pattern of cancellous bone shown in Figure 5. The X-ray skull of a 50+ year female type features in AP shown in Figure 6, and the lateral view showed a widening of the diploic spaces 8-10 mm with ill-defined hyperdense areas in the frontal region (Figure 7). 

**Figure 8:**
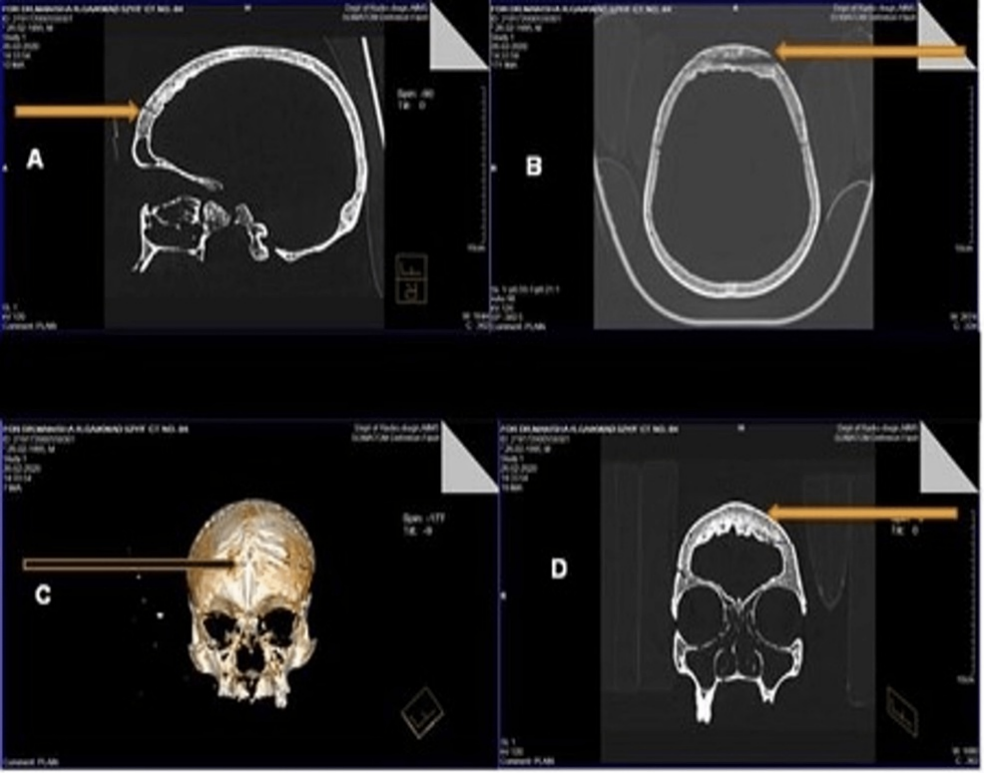
NCCT of the skull revealed widening of the diploic space (diffuse) 9 mm to 12 mm (A); Ill-defined hyperdense lesions in the inner table of the skull (B), thickening of the skull in frontal bone is evidence suggestive of hyperostosis interna of the inner table of the skull in the frontal region (C), Hyperdense lesions surrounding the frontal sinus (D).

The rest of the bony features were normal. NCCT of the skull with bony cuts of 0.1mm skull done with 3D reformat revealed widening of the diploic space (diffuse) 9 mm to 12 mm and ill-defined hyperdense lesions in the inner table of the skull, causing thickening of the skull in frontal bone suggestive of hyperostosis interna of the inner table of the skull in the frontal region shown in Figure 8. 

## Discussion

HFI is present in 5-12% of the population but is almost 9 times more commoner in women than in men, with 40-60% of the cases in post-menopausal women [6,7]. In women, it is an age-related and age-progressive disorder in which extensive proliferation starts after the age of 40 and most frequently after 60 years of age, unlike the males who have shown minimal thickening at the same age [2]. However, it can also be associated with male hypogonadism, atrophy of the testis, and males undergoing complete androgen blockade therapy, which clarifies that the constant exposure to estrogen is one of the primary reasons behind this phenomenon [8]. It has been observed that before the nineteenth and twentieth centuries, there was less prevalence of HFI which contributed to less life span during that time. Thus, human longevity in recent eras, the microevolution of human endocrine regulation by sex steroids, and lifestyle changes like obesity, Type 2 Diabetes Mellitus have also contributed to the increased prevalence of HFI [9,10].

Usually, the growth in HFI is bilaterally symmetrical, with sparing of the regions like the sagittal sinus area, meningeal arteries, and suture lines [2]. The bone encountered in the present study was the frontal bone. The most common hormonal target has been the frontal bone, as per other studies. The presence of Fibroblast Growth Factor (FGF) ligands and 1, 2, and 3 receptors in the frontal bone coupled with the Wnt signaling pathway leads to the increased capacity of bone regeneration and calvaria regrowth [11]. HFI, according to the criteria proposed by Littlejohn [12], can be grouped into 4 types, as shown in Table 1. 

**Table 1:** Littlejohn's Criteria Littlejohn's Criteria [12]

Category	Features
Nil (N):	No new bone formation.
Mild (M):	Early endosteal bone on the inner table of the skull.
Moderate (Mo):	More advanced endosteal bone with bosselated appearance.
Severe (S):	Changes extending beyond the midline with much irregularity and increased thickness.

In some instances, the extension of the HFI is in the dura and falx cerebri. The presence of the effect of estrogen is crucial to the development of HFI. 2 cases of HFI have been found in patients with Klinefelter's syndrome. Kallman syndrome is also associated with HFI [13]. Patients with androgen suppression therapy, like those treated for prostatic cancer, are at greater risk for developing HFI [14]. Famous singer Farinelli who was castrated to preserve his voice, also showed HFI that intensified in an androgen-free environment [15]. 

A study of skulls belonging to the bronze era in Qatna, Syria, has revealed the presence of HFI in tombs of people belonging to the socioeconomically high class. The probability of an increasingly sedentary lifestyle and high-calorie intake could lead to metabolic imbalances leading to HFI, showing that social status can be an indicative marker for the disease [16].

With voluminous literature worldwide on HFI, it has even made its way into a separate entity of metabolic craniopathy; however, its distinctiveness depends on its varied and isolated presentation. Studies have shown that frontal bone thickness > 10 mm is suggestive of HFI and can show reduced tissue oxygenation in the brain (rSO2), causing interference in the cerebral oximetry signals [17].

HFI can cause compression of the dorsolateral frontal cortex causing significant cognitive impairment, including deficits in self-ordered working memory tasks [18]. HFI often has nonspecific and benign symptoms. However, in severe cases, widespread clinical implications starting from headache, motor aphasia, parkinsonism, and depression have also been reported. A rare case of posterior fossa stroke with vertebrobasilar insufficiency, tonsillar herniation, posterior cerebral artery ischemia, and intracranial hypertension requiring craniotomy and cranioplasty has also been found [19].

## Conclusions

In the Indian population, fewer cases of HFI have been reported, indicating a possible distribution variation in different races warranting more studies. Remembering this diagnosis as a helpful adjunct in clinical evaluations is also essential.
